# The value of uterine oncological surgery in a University Hospital. Results of a break-even analysis

**DOI:** 10.1093/eurpub/ckac131.285

**Published:** 2022-10-25

**Authors:** ML Specchia, G Arcuri, A Di Pilla, E La Gatta, T Osti, P Limongelli, G Scambia, RDA Bellantone

**Affiliations:** 1 Fondazione Policlinico Universitario Agostino Gemelli IRCCS, Rome, Italy; 2 Università Cattolica del Sacro Cuore, Rome, Italy

## Abstract

**Background:**

Robotic surgery has many clinical advantages but high costs, raising the issue of healthcare sustainability. This study aims to a comparative analysis of the value, in terms of costs and outcomes, of robotic, laparoscopic, and laparotomy surgery for uterine cancer in a University Hospital.

**Methods:**

An observational retrospective study was carried out on hospitalizations between 1 Jan 2019 and 31 Oct 2021 for uterine cancer surgery. DRG amount, costs, economic margins and 30-days readmissions percentage (mean values and 95% CIs) were calculated for robotic, laparoscopic and laparotomy surgery. Student’s t and Chi-square tests were used to assess differences and the break-even point was calculated.

**Results:**

1336 hospitalizations were analyzed, 366 with robotic, 591 with laparoscopic, and 379 with laparotomy surgery. Robotic surgery compared to laparoscopic and laparotomy ones showed a significant difference (p < 0,001) for economic margin, which was largely negative (-1069.18 €; 95%CI: -1240.44 - -897.92 €) mainly due to devices cost (3549.37 €; 95%CI: 3459.32 € - 3639.43 €), and a lower 30-days readmissions percentage (1.4%; 95%CI: 0.2% - 2.6%) with a significant difference only versus laparotomy (p = 0.029). Laparoscopic compared to laparotomy surgery showed a significantly (p < 0,001) more profitable economic margin (1692.21 €; 95%CI: 1531.75 € - 1852.66 €) without a significant difference for 30-days readmissions. The break-even analysis showed that, on average, for every uterine cancer laparoscopic elective surgery, 1.58 elective robotic surgeries are sustainable for the hospital (95% CI: 1.23 - 2.06).

**Conclusions:**

The systematic application of the break-even analysis will allow defining over time the right distribution of robotic, laparoscopic and laparotomy surgeries’ volumes to perform in order to ensure both quality and economic-financial balance and therefore value of uterine oncological surgery in the University Hospital.

**Key messages:**

• The value-based healthcare approach, defined as the measured improvement in a patient’s health outcomes in relation to its cost, finds effective application in uterine cancer surgery.

• The use of the break-even approach allows to promote the value-based view by identifying a useful criterion for the planning and governance of interventions for uterine malignancies.

